# Understanding, Explanation, and Active Inference

**DOI:** 10.3389/fnsys.2021.772641

**Published:** 2021-11-05

**Authors:** Thomas Parr, Giovanni Pezzulo

**Affiliations:** ^1^Wellcome Centre for Human Neuroimaging, Queen Square Institute of Neurology, University College London, London, United Kingdom; ^2^Institute of Cognitive Sciences and Technologies, National Research Council, Rome, Italy

**Keywords:** active inference, explainable AI, insight, decision making, generative model, understanding

## Abstract

While machine learning techniques have been transformative in solving a range of problems, an important challenge is to understand why they arrive at the decisions they output. Some have argued that this necessitates augmenting machine intelligence with understanding such that, when queried, a machine is able to explain its behaviour (i.e., explainable AI). In this article, we address the issue of machine understanding from the perspective of active inference. This paradigm enables decision making based upon a model of how data are generated. The generative model contains those variables required to explain sensory data, and its inversion may be seen as an attempt to explain the causes of these data. Here we are interested in explanations of one’s own actions. This implies a deep generative model that includes a model of the world, used to infer policies, and a higher-level model that attempts to predict which policies will be selected based upon a space of hypothetical (i.e., counterfactual) explanations—and which can subsequently be used to provide (retrospective) explanations about the policies pursued. We illustrate the construct validity of this notion of understanding in relation to human understanding by highlighting the similarities in computational architecture and the consequences of its dysfunction.

## Introduction

How would we know whether a machine had understood why it chose to do what it did? Simplistically, we might expect that, when queried, it would be able to communicate an explanation for its actions. In this article, we take this to be our operational definition of *machine understanding* (Yufik, 2018). Based on this definition, we can break the problem down into two parts. The first is that a machine must be able to infer why it has taken the actions it has. The second is that it must be able to act to communicate this inference when queried. In thinking about the first—explaining behaviour—it is useful to think about how we go about explaining anything. In the philosophy of science, there is considerable debate about the notion of explanation (Craik, 1952; Bird, 1998; Psillos, 2002), which is beyond the scope of this article. Our use of the term is largely coherent with the idea of “inference to the best explanation” that is common in Bayesian treatments of perception (Helmholtz, 1866; Gregory, 1980) and in philosophy (Lipton, 2017) and proceeds as follows. As scientists, we formulate a series of alternative hypothetical explanations. Each hypothesis entails different predictions about the data that we have measured. By comparing our predictions with those data, we assess which hypothesis is most congruent with our measurements. Translating this same process to explaining behaviour, the implication is that we need a space of hypotheses representing reasons^1^ for behaviour, each of which predicts an alternative course of action. The process of explaining our actions^1^—i.e., having insight into our decisions—then becomes an inference problem. Given some observed sequence of choices, which explanations best fit those data?

This inferential perspective on decision-making is central to active inference (Friston et al., 2014; Parr et al., 2022), which frames perception and action as dual mechanisms that jointly improve our inferences about the causes of our sensory data. While perception is the optimisation of our beliefs to better fit the data we observe, action changes the world to better fit our beliefs. When the internal models required to draw these inferences are temporally deep (Friston et al., 2021), they must include the consequences of the sequential decisions we make while engaging with our environment. Active inference offers a set of prior beliefs about these decisions that represent explanations for behaviour. These explanations divide into three types (Da Costa et al., 2020). First, we select decisions whose sensory consequences cohere with the data anticipated under our model (Åström, 1965; Pezzulo et al., 2018). Second, our choices provide us with data that resolve our uncertainty about our environment (Mirza et al., 2018). Third, the context in which we find ourselves may bias us towards some actions and away from others (Pezzulo et al., 2013; Maisto et al., 2019). The first of these prompts us to head to a restaurant when our internal model predicts satiation when we feel hungry. The second leads us to survey the menu, to resolve our uncertainty about the food on offer. The third biases us towards ordering the same meal as on previous visits to the restaurant. Together, these account for exploitative (preference-seeking), explorative (curiosity-driven), and (context-sensitive) habitual behaviour. The last of these turns out to be particularly important in what follows, as it allows us to construct a narrative as to why we make the choices we do.

In what follows, we consider a simple, well-validated, task that incorporates both explorative, exploitative, and context-sensitive elements (Friston et al., 2015; Chen et al., 2020). It is based upon a T-maze paradigm, in which we start in the centre of the maze. In either the left or the right arm of the T-maze, there is a preferred (i.e., rewarding) stimulus whose position is initially unknown. In the final arm, there is a cue that indicates the location of the rewarding stimulus. To solve this maze and find the reward, we must decide whether to commit to one of the potentially rewarding arms or to seek out information about which is most likely to be profitable before exploiting this information. The twist here is that, after exposure to the maze, we follow up with a query. This takes the form of an instruction to explain either the first or the second move made. By communicating the reason for the action taken, the agent demonstrates a primitive form of insight into their own behaviour.

This touches upon questions about insight into our actions. This concept is important in many fields, ranging from metacognition (Fleming and Dolan, 2012) to cognitive neurology (Ballard et al., 1997; Fotopoulou, 2012) and psychiatry (David, 1990), where some syndromes are characterised by a patient exhibiting a lack of insight into their own behaviour. However, the term “insight” is often used to mean subtly different things and it is worth being clear upon the way in which we use the word here. Note that this is distinct from insight in the sense of the “aha moment”—where a different way of thinking about a problem leads to a clearer understanding of its solution (Kounios and Beeman, 2014; Friston et al., 2017a). In this article, we refer to insight of a different sort. Specifically, how do we come to understand the reasons for our own decision making? To the extent that veridicality is a useful concept here, insight can be regarded as a veridical inference about the causes of behaviour.

The hypothesis implicit in this article is that insight is confabulation, but that this confabulation may be constrained by sensory data to a greater or lesser extent. This provides a behavioural complement to the idea that perception is constrained hallucination (Paolucci, 2021). More precisely, both perception and explanation are inferences. In the extreme case that they are not constrained by data, we call them hallucination or confabulation, respectively. This perspective is endorsed by the philosophical position that, just as we must draw inferences about why other people behave the way they do, our explanations for our own behaviour are also inferred (Carruthers, 2009, 2011). However, we can go further than this. Interestingly, our retrospective (or confabulated) explanations are not innocuous but can change our beliefs about what we did and why. Specifically, hearing our own explanations provides further evidence for the policies we reported, which therefore become more plausible. This suggests an adaptive role for insight in improving our decision making, in addition to the benefits of being able to communicate explanations for behaviour to others.

In what follows, we briefly review the notion of a generative model and active inference. We then outline the specific generative model used throughout this and illustrate the behaviour that results from its solution through numerical simulations. Finally, we offer a summary of the results, in addition to a discussion of the relationship between the structure of these inferences in relation to the neuroanatomy of human cognition.

## The Generative Model

Under active inference, the generative model plays a central role in accounting for different sorts of behaviour. It is the implicit model used by a brain (or synthetic analogue) to explain the data presented by the environment. However, it is more than this. It also represents beliefs about how the world should be—from the perspective of some (biological or synthetic) creature (Bruineberg et al., 2016; Tschantz et al., 2020). This means the generative model guides both a creature’s perception and its actions. Formally, fulfilling these objectives requires scoring the quality of the model as an explanation of data and the quality of the data in relation to the model. The two qualities may be scored using a single objective function: the marginal likelihood or Bayesian model evidence. Simply put, the marginal likelihood scores the probability of observing some measured data given the model. That this depends upon both model and data implies it can be maximised either by modifying the model or by acting to change the data.

In practice, the marginal likelihood is often very difficult to compute. However, it can be approximated by a negative free energy functional (a.k.a., an evidence lower bound or ELBO). This free energy is constructed in relation to a variational (approximate posterior) distribution that maximises the free energy when it is as close as possible to the posterior probability of the hidden states in a model given measured data (Beal, 2003; Winn and Bishop, 2005; Dauwels, 2007). Some accounts of neuronal dynamics rest upon the idea that the activity in populations of neurons parameterises this variational density, and that the evolution of this activity ensures the alignment between the variational and exact posterior distributions (Friston and Kiebel, 2009; Bogacz, 2017; Parr et al., 2019; Da Costa et al., 2021). This means the role of a generative model in active inference is as follows. It determines the dynamics internal to some system (e.g., neuronal dynamics in the brain), and actions that result from these dynamics, *via* a free energy functional that approximates the marginal likelihood of the model. The maximisation of a marginal likelihood is sometimes characterised as “self-evidencing” (Hohwy, 2016).

**Figure 1:**
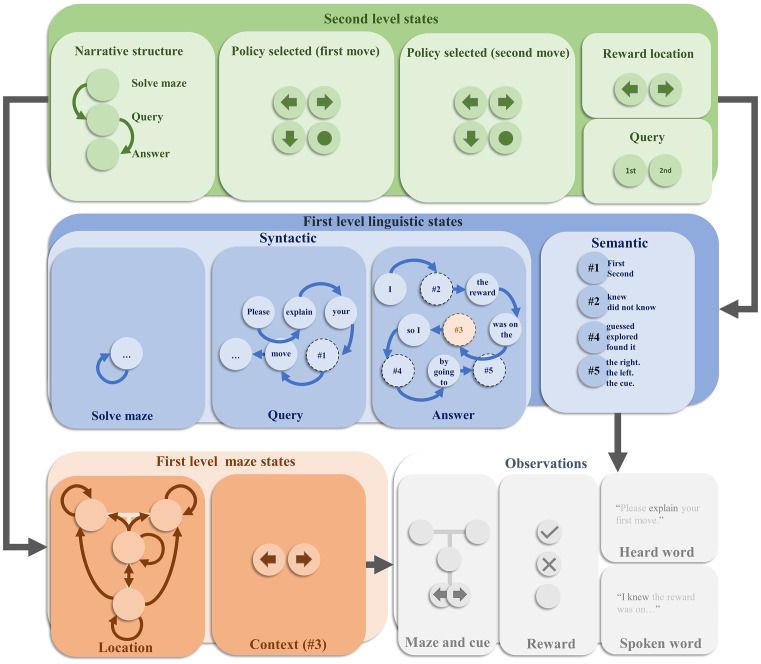
**The generative model**. This schematic offers an overview of the internal model used by an agent to explain how hidden states conspire to generate observable outcomes. This figure is displayed in four main parts. These are the second level hidden states, the first level linguistic states, the first level maze states, and the observed outcomes. Each of these is further decomposed or factorised. The overall structure of the model means that second level states predict first level states. Although not shown here explicitly, the second level states additionally predict the policy (or trajectory) of the location states at the first level, providing a context sensitive bias for decision making. The first level states then combine to predict the observations. Arrows between states within each factor represent allowable transitions. In the absence of arrows, the assumption is that there are no dynamics associated with that state—i.e., it stays the same over time. Prior preferences are attributed to the outcomes such that the central location of the maze is mildly aversive. The reward outcome modality includes an attractive, aversive, and neutral outcome. Please see the main text for more detail.

We now turn to the specific generative model employed in this article. This is depicted in Figure 1. It is a deep temporal model (Friston et al., 2017b), in the sense that it evolves over two distinct timescales. Each level factorises into a set of factors [reminiscent of the idea of neuronal packets (Yufik and Sheridan, 1996)] that simplify the model—in the sense that we do not need to explicitly represent every possible combination of states (Friston and Buzsaki, 2016). At the faster (first) level, the model factorises into maze states and linguistic states. The former describes a T-maze in terms of two state factors (Friston et al., 2015). These are the agent’s location in the maze, and the context—i.e., whether the reward is more likely to be in the left arm or the right arm. The location is controllable by the agent, in the sense that transitions between locations from one timestep to the next depend upon the choices it selects. These alternative transitions are indicated by the arrows in the location panel. Note that the left and right arms are absorbing states—meaning that once entered, the agent cannot leave these locations. In contrast, the context stays the same over time and cannot be changed through action. The allowable policies that the agent can select between are characterised in terms of sequences of actions (i.e., transitions). The first two moves across all the policies cover every possible combination of two moves (transitions to a given location), recalling that the absorbing states ensure that if the first move is to go to the left or right arm, the second move must be to stay there. The maze states predict two outcomes. The first is an exteroceptive outcome, that indicates where the agent is in the maze and, if at the cue location, whether the reward is most likely in the left or the right arm.

The second outcome modality pertains to the reward. Under active inference, there is nothing special about a reward modality: it is treated like any other observation. However, all outcome modalities can be assigned prior probability distributions that specify how likely we are to encounter the different outcomes in that modality. For instance, the generative model employed by a mouse might assign a relatively high prior probability to encounter cheese, in virtue of the fact that mice will act in such a way that they obtain cheese. For this reason, these prior probabilities can be regarded as prior preferences. A rewarding outcome is then simply a preferred or anticipated outcome. In other words, an outcome is rendered rewarding by the agent’s anticipation of encountering it—and its actions to fulfil this expectation.

In our generative model, we include three levels of reward. The first is the attractive outcome (the reward) which is assigned a high relative prior probability. The second is an aversive outcome, which is assigned a low prior probability such that our agent believes it will act to avoid encountering it. The final outcome is a neutral outcome, with an intermediate prior preference. Depending upon the context, the attractive or aversive outcomes are encountered in the left and right arms of the maze, with the neutral outcome found elsewhere. The construction of the maze states is identical to that presented in previous articles, including (Friston et al., 2015, 2017; Chen et al., 2020).

The linguistic states are involved in determining the sentences that will be heard when the behaviour is queried or when responding to the query (i.e., the heard word and spoken word outcome modalities in Figure 1, respectively). As in previous applications of active inference to linguistic communication, these states factorise into syntactic structures and the semantics that can be expressed through this syntax (Friston et al., 2020). The syntactic states take the form of words and placeholder words associated with a set of transition probabilities—which determine which word (or placeholder) follows each other word. For instance, the word “Please” is followed by the word “explain.” Depending upon the first word in the sequence, different syntactic structures appear. If we start with the word “Please”, the syntax is consistent with a query. If starting with the word “I”, it is an answer. In addition, there is a silent syntactic state associated with solving the maze. When the syntactic state is anything other than this silent state, the maze outcomes are set to be in the central location with a neutral reward. This precludes maze-solving (i.e., navigational) behaviour while the agent is attempting to explain its behaviour; and can be regarded as a form of sensory attenuation—as the maze states are functionally disconnected from their associated outcomes during the explanation. The semantic states are the words that can be slotted into the placeholders in the syntactic sequences to provide a meaningful sentence. The third semantic state doubles as the contextual state for the maze.

The slower (second) level deals with the narrative structure of the task, and the maintenance of the information required for its solution. This includes a set of narrative states indicating whether the task is to solve the maze, listen to a query, or respond to that query. These are associated with a prior belief that the first thing to do is to solve the maze and that this is followed by the query and then the response. The narrative states predict the first syntactic state of the sequences at the first level. Specifically, the silent syntax is predicted when the maze should be solved, the syntax beginning “Please” when the query is offered, and the syntax beginning “I” when the answer is required. In addition, the policy is represented at the second level, decomposed into the first and second moves. Each combination of these predicts an alternative policy at the first level. The reward location state predicts the first level context, and the query state predicts whether the first level semantic state associated with the query syntax is “first” or “second”—i.e., whether the query is about the first or the second move. Combinations of these states predict different combinations of semantic states at the first level. For instance, when the narrative state is “answer”, the query state is “first”, and the first move state is a move to the cue location, the second semantic state is predicted to be ‘did not know’, the fourth semantic state is predicted to be “explored”, and the fifth semantic state is predicted to be “the cue”.

We will not unpack the details of the solution to this form of the generative model here, as they have been detailed in numerous other publications (Friston et al., 2017, b; Parr et al., 2019; Da Costa et al., 2020; Sajid et al., 2021). However, we provide a brief outline of the procedure. In short, the generative model outlined above can be formulated in terms of a joint probability distribution over the states (*s*^(*i*)^) at each level (indicated by the superscript), the policy at the first level (*π*^(1)^), and the outcomes they generate (*o*). The marginal likelihood of this model can be approximated by a negative free energy functional (*F*) which can be recursively defined as follows:


$$F^{\left(2\right)}\;\left(o\right)\;=\;D_{KL}\;\left[Q\left(s^{\left(2\right)}\right)\;||\;P\;\left(s^{\left(2\right)}\right)\right]\;+\;E_{Q\left(s^{\left(2\right)}\right)}\;\left[F^{\left(1\right)}\left(o,s^{\left(2\right)}\right)\right]$$

$$F^{\left(1\right)}\;\left(o,s^{\left(2\right)}\right)\;=\;D_{KL}\;\left[Q\left(\pi^{\left(1\right)}\right)\;||\;P\left(\pi^{\left(1\right)}\;|s^{\left(2\right)}\right)\right]\;+\;E_{Q\left(\pi^{\left(1\right)}\right)}\;\left[F^{\left(1\right)}\;\left(o,\pi^{\left(1\right)},s^{\left(2\right)}\right)\right]$$

(1) $$F^{\left(1\right)}\;\left(o,\pi^{\left(1\right)},s^{\left(2\right)}\right)\;=\;D_{KL}\;\left[Q\left(s^{\left(1\right)}|\pi^{\left(1\right)}\right)\;||\;P\left(s^{\left(1\right)}\;|\pi^{\left(1\right)},s^{\left(2\right)}\right)\right]\;-\;E_{Q\left(s^{\left(1\right)}|\pi^{\left(1\right)}\right)}\;\left[\ln\;P\left(o|s^{\left(1\right)}\right)\right]\;(1)$$

In Equation 1, the E symbol means “expectation” or average. The *Q* distributions are the variational distributions that approximate posterior probabilities and the symbol *D_KL_* represents a Kullback-Leibler divergence—which quantifies how different two probability distributions are from one another. Beliefs about each set of states and policies in the model are computed as follows:


$$Q\left(s^{\left(2\right)}\right)\;=\;\underset{Q\left(s^{\left(2\right)}\right)}{\arg\;\min}\;F^{\left(2\right)}\;\left(o\right)$$

$$Q\left(\pi^{\left(1\right)}\right)\;=\;\underset{Q\left(\pi^{\left(1\right)}\right)}{\arg\;\min}\;E_{Q\left(s^{\left(2\right)}\right)}\;\left[F^{\left(1\right)}\;\left(o,s^{\left(2\right)}\right)\right]$$

(2) $$Q\left(s^{\left(1\right)}|\pi^{\left(1\right)}\right)\;=\;\underset{Q\left(s^{\left(1\right)}|\pi^{\left(1\right)}\right)}{\arg\;\min}\;E_{Q\left(s^{\left(2\right)}\right)}\;\left[F^{\left(1\right)}\;\left(o,\pi^{\left(1\right)},s^{\left(2\right)}\right)\right]\;(2)$$

The second line depends upon the empirical (conditional) prior probability for each policy. This is given as:


(3) $$\ln\;P\;\left(\pi^{\left(1\right)}|s^{\left(2\right)}\right)\;\alpha \;\ln\;E\;\left(\pi^{\left(1\right)}|s^{\left(2\right)}\right)\;-\;G\;\left(\pi^{\left(1\right)}|s^{\left(2\right)}\right)\;G\left(\pi^{\left(1\right)}|s^{\left(2\right)}\right)\;G\;\left(\pi^{\left(1\right)}|s^{\left(2\right)}\right)\;\underset{=}{\Delta }\;D_{KL}\;\left[Q\left(o|\pi^{\left(1\right)}\right)\;||\;P\left(o|C\right)\right]\;+\;E_{Q\left(s^{\left(1\right)}|\pi^{\left(1\right)}\right)}\;\left[H\left[P\left(o|s^{\left(1\right)}\right)\right]\right]\;(3)$$

Here, *E* is a function that acts as a prior weight or bias—conditioned upon the second level states, for the policies (Parr et al., 2021). The *H* in the second line is a Shannon entropy and *C* parameterises the preferred outcomes. The function *G* is referred to as expected free energy, and penalises those policies associated with large deviations from preferred outcomes, and policies in which the outcomes are uninformative about the hidden states.

This generative model permits two different types of action. These can be distinguished based upon how they influence the outcomes. The first sort of action influences the hidden states, which then cause changes in outcomes. Movement from one location to another in the maze falls under this category. In practice, these actions are chosen based upon the policy inferred to be the most probable *a posteriori*. The second sort of action directly influences the outcomes. This is the form of action involved in generating the linguistic outcomes (specifically, the spoken word). The latter are selected to minimise the free energy given current beliefs:


(4) $$o_{\tau +1}\;=\;\underset{o_{\tau +1}}{\arg\;\min}\;F^{\left(2\right)}\;\left(o_{\tau +1}|o_{t\leq \tau }\right)\;=\;\underset{o_{\tau +1}}{\arg\;\max}E_{Q\left(s^{\left(1\right)},\pi^{\left(1\right)}\right)}\;\left[\ln P\left(o_{\tau +1}|s_{\tau +1}^{\left(1\right)}\right)\right]\;(4)$$

This is in the same spirit as formulations of active inference in terms of predictive coding with reflexes. The idea is that by predicting the data we would anticipate given our beliefs, low-level reflexes of the sort found in the spinal cord or brainstem can correct deviations between our predictions and measurable data such that our predictions are fulfilled (Adams et al., 2013; Shipp et al., 2013). Having outlined the generative model, and the principles that underwrite its solution, we next turn to a series of numerical simulations that demonstrate some of the key behaviours of this model.

## Simulations

In this section, we attempt to do three things. First, we illustrate the behaviour of an agent who relies upon the generative model outlined in the preceding section. We then attempt to offer some intuition as to the belief updating that underwrites this behaviour, and in doing so highlight the belief updating that occurs over multiple timescales in deep temporal models of this sort. In addition, we demonstrate the emergence of replay phenomena—of the sort that might be measured in the hippocampus of behaving rodents. Finally, we investigate what happens when we violate the assumptions of the generative model and the confabulatory explanations that result.

**Figure 2:**
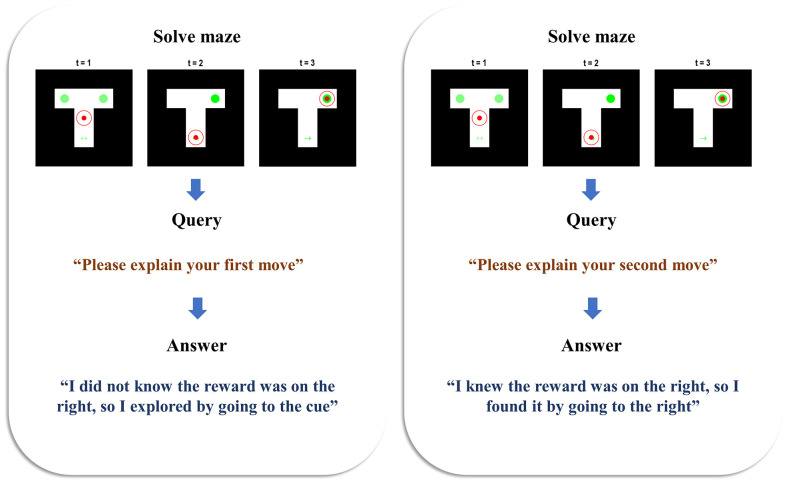
**Beliefs and behaviour**. This figure reports the results of two simulations—one in which the first move is queried (left), and one in which the second move is queried (right). Each is divided into the three stages of the task. The first row of plots shows the beliefs and behaviour as the maze is solved during the first three time-steps. The small filled red circle shows the position of the agent in the maze. The larger unfilled circle shows the beliefs of the agent about its position. When this is red, this indicates a posterior probability of one of being in that location. As the probability decreases, the red fades to nothing—i.e., a posterior probability of zero of being in that location. In both simulations, the agent maintained confident and veridical beliefs about its location. The beliefs about the context (i.e., reward-left and reward-right) are shown in green, with the intensity of the green colour in the left and right arms (and of the arrows in the cue location) corresponds to the probability assigned to the associated context under the agent’s posterior beliefs. On solving the maze, the agent moves on to the query stage, and the sentence presented to the agent is shown. Finally, the agent has the opportunity to answer the query, and the sentence it generates is shown below.

Figure 2 illustrates the behaviour and belief updating that occurs during the maze task, followed by the query presented to the agent and the response it offers. Two simulations are presented to show the answers given to two different queries, following the same behaviour. In both cases, the agent is initially uncertain about the context, as shown by the faint green circles in the left and right arms—indicating an equal posterior probability assigned to the reward being on the left or right. The agent starts in the central location and maintains veridical beliefs about its location throughout. At the second timestep, we see that the agent has elected to explore, seeking out the cue arm. On observing a cue indicating the right context, it updates its beliefs such that the reward is now anticipated in the right arm. At the third timestep, it has moved to the right arm, finding the reward there. When queried about the reasons for the first move, the agent sensibly replies that it did not know where the reward was (as we can verify from the plot of the maze at *t* = 1), so it explored by going to the cue location (as we can verify from the maze plot at *t* = 2). When queried about the second move, it replies that it did know where the reward was (again, verifiable from the maze plot at *t* = 2)—having already seen the cue by this point—and that it consequently went to find the reward in the right arm. This pair of simulations illustrates that the generative model is sufficient for the agent to infer the actions it has taken, to come to a reasonable explanation of the motivations behind these actions, and to explain this when queried. In accordance with our definition in the introduction, this meets the criteria for a (simple) form of understanding.

**Figure 3:**
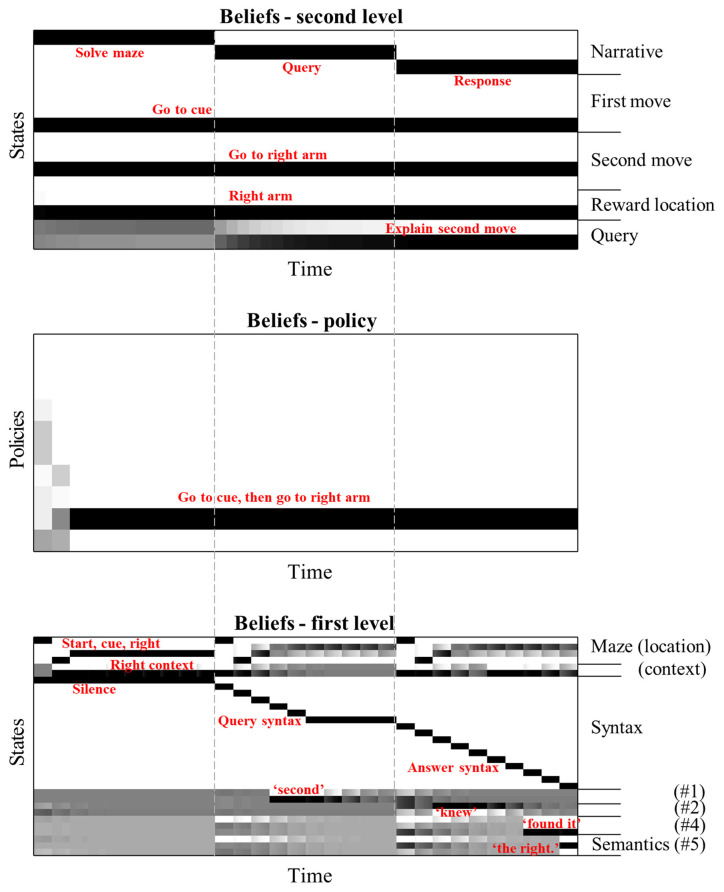
**Hierarchical belief updating**. This figure shows the beliefs about states and policies over time in the temporally deep model. The main message of this figure is that this updating occurs over distinct timescales, with the first level states being updated much faster than those of the second level. The layout of these plots are as follows. Each row within each plot represents an alternative state or policy. The *x*-axis represents time; such that columns of the plot are discrete time steps. The shading of each cell in the state (or policy) x time arrays indicates the posterior probability assigned to that state (or policy) at that time. Black is a probability of one, white of zero, with intermediate shades representing intermediate probabilities. To avoid overcrowding, we have not labelled each row individually, but have annotated the states (and policies) that are inferred to be most likely in red. The vertical dashed lines indicate the alignment of the three epochs at the slowest (second level) with the inferences about the policies and states. Interpreted from a computational neuroscience point of view, each row of each plot can be regarded as a raster plot, indicating the aggregated firing rates of a distinct population of neurons.

To delve into the mechanisms by which this understanding is achieved, Figure 3 details the beliefs held by the agent about the variables in the generative model throughout the simulation from the right of Figure 2. The grey dashed lines indicate the timesteps at the slower (second) level of the model, referred to hereafter as “epochs”, and illustrate their alignment with the time-course of the faster) first level. During the first epoch, we see the first level beliefs (lower panel) being updated in accordance with the solution to the maze in Figure 2. The sequence here is reminiscent of the sequential activation of hippocampal place cells as rodents move through a series of locations (O’Keefe and Dostrovsky, 1971; Foster and Wilson, 2007). Inferences about the semantic states (i.e., the words #1 to #5 shown at the bottom of the bottom panel) remain uncertain during this time. Note the update in beliefs about the context (see “Right context” in the lower panel) on reaching the cue location, as the agent obtains the cue and goes from believing both contexts to be equally likely to believe that the right context is in play. The accompanying belief-updating for the policy (centre panel) shows that initially, the agent believes it will choose one of the many policies that start by going to the cue location, which correspond to the rows coloured in grey —consistent with the epistemic affordance associated with this location. On reaching the cue location, all uncertainty about the context is resolved, meaning the only remaining motivational drive is to obtain the cue. This prompts further belief updating about the policy, favouring the single policy in which the first move was to go to the cue and the second to the right arm. On enacting this policy and receiving the associated sensory input (i.e., observing itself going to the cue then right arm locations), the agent becomes confident that this is the policy it has pursued.

The inferred policy and context now allow for updating of beliefs about the first epoch at the second level. Practically, the updating of beliefs at each level happens asynchronously in this implementation, such that beliefs at the second level are updated following the updates at the first level. This asynchronous updating rests upon an adiabatic assumption, which means the two timescales in question may be treated under a mean-field assumption (i.e., approximately independently of one another). Consistent with the first level inferences, the second level beliefs over this epoch are updated such that the first and second moves inferred are consistent with the selected policy, and the reward location is consistent with the maze context. These beliefs are then used to provide empirical priors for the first level during the second epoch. Note the second epoch begins with a veridical belief about the policy selected and the maze context—ensuring these do not have to be re-inferred by the first level.

During the second epoch at the first level, the query is presented to the agent. Once the word “second” is heard, it is able to update its belief about the first semantic state. At the end of the epoch, this is propagated to the second level, allowing for belief updating so that the query is inferred to be about the second move. This belief about the query is propagated through to the third epoch by the second level, again providing an empirical prior belief to the first level that the second query must now be answered. In addition, beliefs about the reward location and the moves selected at the second level combine with the belief about the query to provide prior beliefs about the semantic states. These beliefs lead the agent to generate the appropriate response to the question.

**Figure 4:**
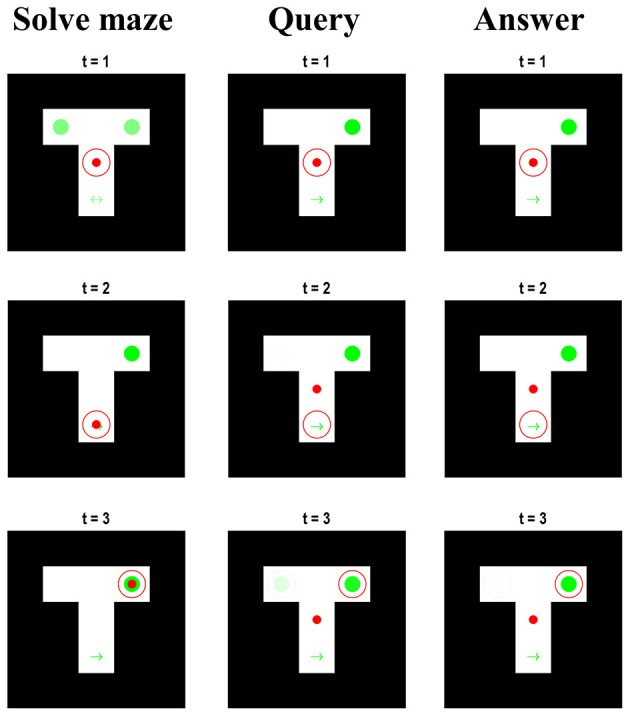
**Maze beliefs and replay**. This figure reports the beliefs of the agent for the first three time-steps during each of the slower epochs at the narrative level. Each column of images displays a single epoch, with each row displaying the beliefs (and location) at each of the first three steps. The format of each image is the same as in Figure 2. These can be regarded as visual displays of the belief updates shown in Figure 3. The important things to note are: (i) that maze-solving epoch is identical to that of Figure 2; (ii) that the true location (i.e., red dot) is central in the query and answer epochs, (iii) that the context is known from the start in the latter two epochs, and (iv) that the beliefs about the location hidden states are consistent throughout all three epochs.

An interesting feature of the belief updating shown in Figure 3 is the updates in beliefs about the maze location during the second and third epochs. Recall that, when the syntactic states are consistent with the query or answer, the maze states are decoupled from the associated outcomes—which are set at the central location and neutral reward. Despite this, the beliefs about the location in the maze during the first few timesteps of the second and third epoch appear to replay the beliefs that were held when solving the maze. Figure 4 examines this more closely, by plotting the beliefs about the maze for the first three timesteps during each epoch. Note that, although the red dot remains in the centre during the query and answer epochs, the red circle indicating the inferred location moves according to the same sequence as in the maze-solving epoch—however, the beliefs about the context are preserved from the end of the maze-solving. Replay of this sort has been identified physiologically in rodents in the same hippocampal cells that signal sequences of locations while behaving (Louie and Wilson, 2001; Foster, 2017; Pezzulo et al., 2017), hinting that the mechanisms that solve this generative model may also be at work in biological brains. That these mechanisms play a functional role is evidenced by the fact that interrupting hippocampal sharp-wave ripple activity (during which replays often occur) impairs memory-guided navigational choices (Jadhav et al., 2012). Interestingly, models built with the aim of simulating replay call upon a similar hierarchical structure in which the highest (narrative) level of the model involves an alternation between behaviour and replay sequences (Stoianov et al., 2021). Although these models focus more upon the role of replay in learning, our simulations suggest that such models can be interpreted, loosely, as if the synthetic agents are attempting to make sense of their previous actions during the replay sequences.

So why does replay occur when solving this model? The answer to this has two parts. In Bayesian statistics, the inference we draw depends upon a prior and a likelihood. In our model, both contribute to the development of replay. Recall that, while the query and answer syntactic states are in play, the maze outcomes are fixed. This means that no matter which actions the agent chooses, it will receive no sensory feedback coherent with those choices. This means the likelihood distribution is rendered uninformative and effectively uncouples the reality of the agent’s position in the maze from the beliefs it has about this location. While this ensures the agent remains—for all practical purposes—fixed to the spot, it also liberates [or detaches (Gärdenfors, 2005; Pezzulo and Castelfranchi, 2007)] the inference process from the constraints of sensory input. As such, it can be seen as a form of sensory attenuation of the sort we might anticipate during dreaming (Windt et al., 2014), imagination (Villena-González et al., 2016; Kilteni et al., 2018), or episodic recall (Conway, 2001; Barron et al., 2020). This accounts for the role of the likelihood. However, freeing the agent from the constraints of sensation is not sufficient for replay. We also need a prior that assigns greater plausibility to the previous sequence of actions. This comes from the second level inference about the actions taken during the first epoch and their propagation to subsequent epochs as empirical priors. In other words, when sensory input is attenuated, a generative model simply recirculates prior information. In our example, this information pertains to the previous sequence of actions, but it could relate to other regularities learned during previous exposure to sensory data (Fiser et al., 2010; Buesing et al., 2011; Pezzulo et al., 2021). It is important to note that this construction was not designed to simulate replay. It is an emergent feature when beliefs about the policy must be propagated forwards in time (i.e., held in working memory) to help answer questions later.

**Figure 5:**
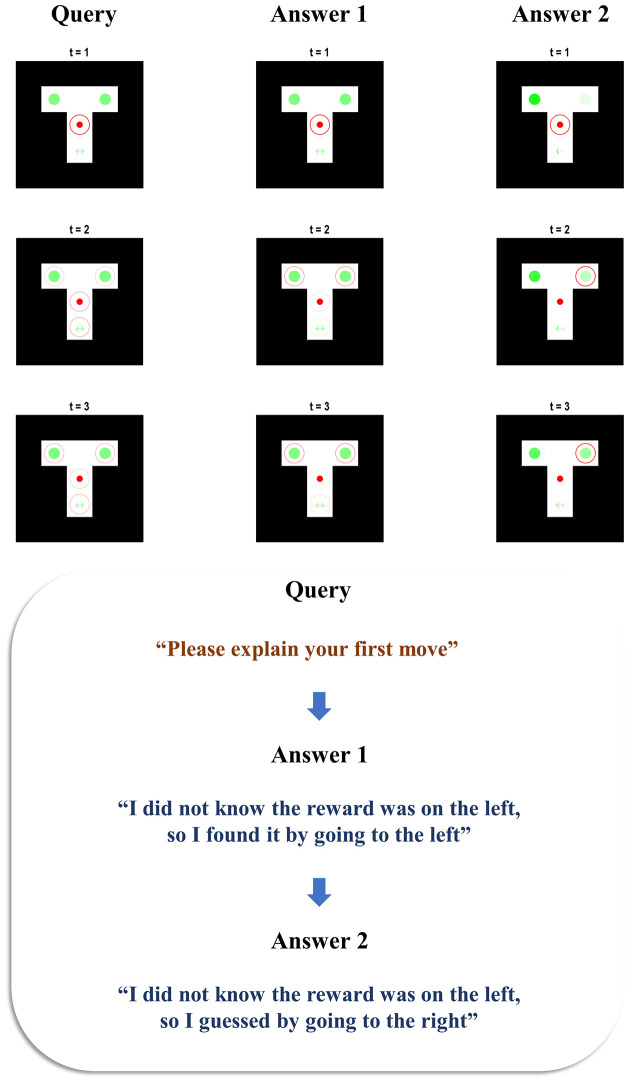
**Confabulation**. This figure reports the behaviour of an agent who confabulates an answer as to why it pursued the course of action that it did, in virtue of never having pursued any course of action. It is given two opportunities to explain itself. The first answer given is nonsensical, as the generative model assumes that going straight to the reward.

Another feature of the belief updates from Figure 2 that is worth unpacking is the increase in confidence about the context during the answer epoch. This seems counterintuitive, as the agent has had no new access to the maze outcomes. However has at the beginning of the maze-solving epoch. We see that, during the query epoch in Figure 5, the agent is uncertain about the state of the maze and the actions it took. It is confident that it started out in the first location and ascribes a slightly higher probability to being away from the central location by the third timestep, consistent with the fact that most plausible policies involve moving away from here. The probability of ending up in the left or right arm increases over time, as these are absorbing states. This in turn lends those policies leading to those states greater plausibility.

The beliefs about the left and right arms are similar during the first answer epoch (see the lower image of the centre column of Figure 5). This is because, by the third timestep of the first answer epoch, the agent has heard itself say that it did not know the reward location but has not yet heard its assertion that the reward was on the left. Taken together, the agent’s first answer does not make much sense. If going straight to the left on the first move, the agent should have known it was on the left in advance. This is even more puzzling when we note that the generative model takes a move that results in the reward location as evidence that the reward location was known. However, the apparent mismatch between not knowing it was on the left, but going straight there to find it anyway, is understandable when we consider that it is the second level of the model that enforces internal consistency in the story told by the agent. In our previous simulations (Figure 2), the agent already has a good idea as to which moves it made and the context of the maze by the time of the query epoch. In Figure 5, the agent is unable to formulate these beliefs until the first time it hears itself giving the explanation. However, by the second answer epoch, it has had a chance to synthesise what it has heard itself saying, and to revise this to an internally consistent explanation. Here, it has taken the fact that it did not know the reward location, and that it was on the left, and inferred that it must have guessed incorrectly given that it did not know the location. The result is the inference that it guessed at the reward location and got it wrong; a perfectly internally consistent, if confabulated, story.

## Discussion

This article was designed to address the problem of machine understanding and to show what this might look like using an active inferential approach in a simple example setting. The solution was based upon a deep temporal model, whose separation into two timescales allowed for a narrative overview of the task, and the propagation of information from one epoch to the next. The separation of timescales inherent to the model, and associated belief updating, in Figure 3 is a generic feature of many deep temporal models. For example, in Friston et al. (2017b) a similar construction was used to simulate reading, where each word in a sentence provides information about the letters in the next word. In (Heins et al., 2020), a deep temporal model was employed for the purposes of a visual search paradigm, where each fixation point was associated with a dot-motion evidence accumulation task (Shadlen and Newsome, 1996). Similar approaches have been employed for working memory tasks (Parr and Friston, 2017), enabling the maintenance of information “in mind” throughout a delay period (Funahashi et al., 1989). These models have also found application in the modelling of emotions (Smith et al., 2019) and “affective inference” (Hesp et al., 2021). They have additionally been formulated through neural network models of the kind found in machine learning (Ueltzhöffer, 2018).

Probably the closest functional homologue to the process in this article was a deep temporal model of motor control (Parr et al., 2021), in which sequences of small movements were composed into longer trajectories *via* a higher (slower) level of the model. As in this article, this called upon the propagation not just of beliefs about states, but of beliefs about policies from one epoch to the next. One of the contributions of the modelling of motor control was to examine the consequences of a lesion to the connection between the two levels. Interestingly, lesions of the generative model for motor control produced a lack of coherence in movement trajectories that is formally analogous to the incoherent story confabulated during the first answer in Figure 5 (later made consistent through the input from the second level).

The functional architecture of the homologous processes in the brain appears to involve the prefrontal cortices. Working memory is a good example of this, as the neural populations exhibiting persistent activity throughout delay-periods have been identified in the prefrontal cortex (Funahashi et al., 1989; Botvinick, 2008). However, these structures have also been linked directly to metacognition—the ability to assess one’s own cognition—*via* lesion studies (Fleming et al., 2014). The frontal cortices interact directly—and *via* subcortical nuclei—with the temporal cortices (Kier et al., 2004; Blankenship et al., 2016; Rikhye et al., 2018), whose lateral surfaces are associated with language (Price, 2000; Hutsler, 2003), and whose medial surfaces are associated with episodic memory and recall (Squire and Zola, 1998; Eichenbaum et al., 2012). They also share dense reciprocal connections with the basal ganglia (Naito and Kita, 1994)—the set of grey-matter nuclei most associated with the adjudication between alternative actions (Nambu, 2004). This hints at homology between the structure of the generative model in this article and the anatomy of the associated neuronal computation, providing a construct validation of our formulation of action understanding in relation to human understanding. It is also interesting to note that confabulatory pathologies in humans (Korsakoff, 1887) arise when the connectivity between the frontal, temporal, and subcortical nuclei are disrupted (Korsakoff, 1887; Benson et al., 1996; Turner et al., 2008), further endorsing the computational anatomy. Conceptually, starting from the query epoch in Figure 5 may be analogous to a disconnection that precludes a memory of the maze-solving epoch from being propagated to the query epoch, such that we arrive at the query epoch as if it were the first epoch.

Given that the primary aim of this article was to address understanding of actions, it is interesting to note that some phenomena that feature in living machines (like us) emerge on solving this problem. The emergence of replay is of particular interest in the context of theories about the emergence of episodic memory. This form of memory has two defining features. The first is that it is declarative, in the sense that its contents can in principle be “declared” (Anderson, 1976; Squire and Zola, 1998). This contrasts with, for example, procedural memories. The second defining feature of episodic memory is that it is associated with a spatiotemporal context—in contrast to semantic memories of facts that may be divorced from such contexts. The replay phenomena shown in Figures 3, 4 meets both these criteria. We know it can be declared, as this is precisely what happens when the query is answered. It is spatiotemporal, in the sense that it is a memory of a sequence of locations in time. As such, one could view this as a simulation of a primitive sort of episodic memory. The reason this is interesting is that one explanation for episodic memory in biological creatures suggests that it developed alongside the ability to communicate past experiences (Mahr and Csibra, 2018). The simulations presented here lend some weight to these ideas, given that we set out to develop a model capable of explaining past actions, and found physiological hallmarks of episodic memory (i.e., replay) in the resulting belief updating. This is not in conflict with the conception of episodic memory as supporting a form of mental time-travel in the past and the future, enabling recall of the past and imagination of the future. As demonstrated in Figure 5, the agent is perfectly capable of using the same machinery for imagination of events that have not yet happened.

The central theme of this article is an inference about “what caused me to do that?” However, the status of Bayesian methods in establishing causation of this sort is controversial. The reason for this is that Bayes’ theorem is symmetrical. It says that the product of a prior and likelihood is the product of a marginal likelihood and posterior probability. However, the labels “prior” and “marginal likelihood” can be swapped (provided “likelihood” and “posterior” are also swapped) without compromising the formal integrity of the theorem. This cautions against interpreting a conditional probability as a causal statement. This is less worrying in our context, as we know by construction that sensory data are caused by hidden states—i.e., we have implicitly built in a causal assumption to the model. However, the role of policies as causes of behaviour is a little more nuanced.

An influential formalism designed to address causality (Pearl, 2009, 2010) rests upon the idea of interventions. Under this formalism, an important notation is the “do” notation, in which *P*(*y*| do(*x*)) is the distribution of *y* once *x* is fixed through some intervention. This breaks the symmetry of Bayes’ theorem as, if *x* causes *y*, *P*(*y*| do(*x*)) will be equal to *P*(*y*| *x*), but *P*(*x*| do(*y*)) will be equal to *P*(*x*). The concept of intervention helps to contextualise the notion of a causal hierarchy—sometimes referred to as “Pearl’s hierarchy” (Bareinboim et al., 2020). This hierarchy distinguishes between the three levels of the generative model. In ascending order, these are associational, interventional, and counterfactual. This provides a useful framework in which to situate the generative model outlined in this article. Given that the relationship between policies and the sequence of states is articulated in terms of conditional probabilities, our generative model must be at least at the associative level of Pearl’s hierarchy. Implicitly, the interventional level criteria are also met, in that the inversion of the model employs a structured variational distribution (Dauwels, 2007) in which the marginals for the first level are evaluated as being independent of the second level states. This means the model is treated as if *P*(*s*^(1)^|*π*^(1)^) is equal to *P*(*s*^(1)^| do(*π*^(1)^)) and *P*(*π*^ (1)^) is equal to *P*(*π*^ (1)^| do(*s*^(1)^)). However, it is worth noting that this applies only to the location states at the first level—the other states being conditionally independent of the policy given the second level states (i.e., the first level explanation is not caused by the policy pursued in an interventional sense, although there is an associational form of causality linking the two). In addition, the second level states do play a causative role, ensuring that the explanation at this level also causes the policy it attempts to account for. The third level of the Pearl hierarchy is more interesting from our perspective, given the emergence of a simple form of imagination as we saw in Figure 5. The criteria for counterfactual causation are met by noting that, initially, beliefs about all policies are evaluated for each policy. For each policy, this means there are a set of beliefs about states as if that policy were pursued. It is this counterfactual inference that facilitates the confabulation observed on asking for an explanation for a policy never pursued.

In this article, we elaborated on an operational notion of understanding as “inference to the best explanation” and described an active inference agent that is able to infer and communicate an explanation for its actions. However, the nature of understanding is a longstanding problem in philosophy—which we make no claims as to having solved. An interesting question is whether our agent (or more broadly, any artificial system) really understands anything. While addressing this question is clearly beyond the scope of this article, we hope that providing an example of an artificial system that appears to understand its actions helps advance the theoretical debate—and assists in the identification of what is still missing from current operational definitions of understanding.

An interesting extension to this work would be to incorporate the response “I don’t know” as an alternative to the explanations available to the agent. We have assumed this is unavailable to the agent in our simulations, as it is reasonable to assume that if we behave a particular way, we believe we know why we did. However, this prompts attempts at explanation despite not having engaged in the task. While it is interesting that these explanations enable the agent to convince itself of what has happened, we might anticipate that an agent could spare itself spurious explanations if able to infer that it is not sure of the answer. This might then point to the mechanisms for confabulation and loss of insight in psychopathology—framing it as a failure of inference about what is and is not known. However, this is not a straightforward problem to solve. This is evidenced by the (metacognitive) difficulties people have in assessing their own ability at performing even the simplest of tasks (Fleming et al., 2010; Fleming, 2021). Another interesting avenue would be to consider the role of two agents communicating with one another on task performance (Bahrami et al., 2012; Shea et al., 2014). For instance, it would be interesting to see whether, on receiving the explanation from an agent who has just completed the task, a second agent may perform the task more efficiently. Furthermore, the choice of question by the second agent may be more interesting, as they may wish to resolve uncertainty not just about the actions of the first agent, but of the structure of the task itself. For an example of this sort of diachronic inference, please see Friston et al. (2020). Diachronic inference refers to inferences drawn when two agents engage in a form of turn-taking, as is common in conversation, giving a periodic switching between speaking and listening. The current article dealt with only a single switch (from listening to speaking), which could usefully be expanded into a more extended conversation.

## Conclusion

A key challenge for machine learning and artificial intelligence is to overcome the problem of understanding. While these approaches have been successful in making a range of decisions, the explanations for these decisions is often opaque. This article has sought to set out what a system capable of understanding and providing explanations for its decisions might look like. We took as our operational definition of understanding the ability to disambiguate between alternative hypotheses as to the reasons for behaving in a particular way and the ability to communicate the inferred reason for this behaviour, on being queried. To this aim, we constructed a generative model that predicts both behaviour and its (linguistic) explanation. This called upon a deep model that propagates information about choices through multiple epochs, enabling the presentation of a task (a simple T-maze), a query epoch, and an answer epoch. We demonstrated that inversion of this model under active inference allows for convincing explanations for the decisions made when solving the task. Interestingly, these explanations can also change our beliefs about what somebody did and why. Furthermore, biological phenomena such as replay emerge from this inversion—affording evidence for theories of episodic memory based upon a need to communicate past events. Finally, we saw that the pathologies of inference—on violation of the assumptions of the model—are similar to those seen in human behaviour in the context of some psychopathologies. The pathological explanations we encountered highlight that understanding can be thought of as constrained confabulation, but that it is constrained to a greater or lesser degree by the quality of the data used to form explanations.

## Data Availability Statement

Publicly available datasets were analysed in this study. This data can be found here: https://www.fil.ion.ucl.ac.uk/spm/software/spm12/.

## Author Contributions

All authors listed have made a substantial, direct and intellectual contribution to the work, and approved it for publication. All authors contributed to the article and approved the submitted version.

## Conflict of Interest

The authors declare that the research was conducted in the absence of any commercial or financial relationships that could be construed as a potential conflict of interest.

## Publisher’s Note

All claims expressed in this article are solely those of the authors and do not necessarily represent those of their affiliated organizations, or those of the publisher, the editors and the reviewers. Any product that may be evaluated in this article, or claim that may be made by its manufacturer, is not guaranteed or endorsed by the publisher.

## Footnotes

^1^Note that not all hypotheses represent reasons for doing something, and the idea of reason does not follow directly from the scientific analogy. Central to this article is the idea that the hypotheses we are interested in can be translated into a verbal explanation that can be recognised as a reason for behaviour.

## References

[B1] Adams R. A. Shipp S. Friston K. J. (2013). Predictions not commands: active inference in the motor system. Brain Struct. Funct. 218, 611–643. 10.1007/s00429-012-0475-5 23129312 PMC3637647

[B2] Anderson J. R. (1976). Language, Memory and Thought. UK: Lawrence Erlbaum.

[B3] Åström K. J. (1965). Optimal control of markov processes with incomplete state information. J. Math. Anal. Appl. 10, 174–205. 10.1016/0022-247X(65)90154-X

[B4] Bahrami B. Olsen K. Bang D. Roepstorff A. Rees G. Frith C. (2012). What failure in collective decision-making tells us about metacognition. Philos. Trans. R. Soc. Lond. B. Biol. Sci. 367, 1350–1365. 10.1098/rstb.2011.0420 22492752 PMC3318766

[B5] Ballard C. McKeith I. Harrison R. O’Brien J. Thompson P. Lowery K.. (1997). A detailed phenomenological comparison of complex visual hallucinations in dementia with lewy bodies and Alzheimer’s disease. Int. Psychogeriatr. 9, 381–388. 10.1017/s1041610297004523 9549588

[B6] Bareinboim E. Correa D. Ibeling D. Icard T. (2020). On Pearl’s Hierarchy and the Foundations of Causal Inference. Columbia CausalAI Laboratory, Technical Report(R-60).

[B7] Barron H. C. Auksztulewicz R. Friston K. (2020). Prediction and memory: a predictive coding account. Prog. Neurobiol. 192:101821. 10.1016/j.pneurobio.2020.101821 32446883 PMC7305946

[B8] Beal M. J. (2003). Variational Algorithms for Approximate Bayesian Inference. United Kingdom: University of London.

[B9] Benson D. F. Djenderedjian A. Miller B. L. Pachana N. A. Chang L. Itti L.. (1996). Neural basis of confabulation. Neurology 46, 1239–1243. 10.1212/wnl.46.5.1239 8628459

[B10] Bird A. (1998). Philosophy of Science. London: McGill-Queen’s University Press.

[B11] Blankenship T. L. O’Neill M. Deater-Deckard K. Diana R. A. Bell M. A. (2016). Frontotemporal functional connectivity and executive functions contribute to episodic memory performance. Int. J. Psychophysiol. 107, 72–82. 10.1016/j.ijpsycho.2016.06.014 27388478 PMC4986699

[B12] Bogacz R. (2017). A tutorial on the free-energy framework for modelling perception and learning. J. Math. Psychol. 76, 198–211. 10.1016/j.jmp.2015.11.003 28298703 PMC5341759

[B13] Botvinick M. M. (2008). Hierarchical models of behavior and prefrontal function. Trends Cogn. Sci. 12, 201–208. 10.1016/j.tics.2008.02.009 18420448 PMC2957875

[B14] Bruineberg J. Kiverstein J. Rietveld E. (2016). The anticipating brain is not a scientist: the free-energy principle from an ecological-enactive perspective. Synthese 195, 2417–2444. 10.1007/s11229-016-1239-1 30996493 PMC6438652

[B15] Buesing L. Bill J. Nessler B. Maass W. (2011). Neural dynamics as sampling: a model for stochastic computation in recurrent networks of spiking neurons. PLoS Comput. Biol. 7:e1002211. 10.1371/journal.pcbi.1002211 22096452 PMC3207943

[B16] Carruthers P. (2009). How we know our own minds: the relationship between mindreading and metacognition. Behav. Brain Sci. 32, 121–138. 10.1017/S0140525X09000545 19386144

[B17] Carruthers P. (2011). The Opacity of Mind: An Integrative Theory of Self-Knowledge. Oxford: Oxford University Press.

[B18] Chen A. G. Benrimoh D. Parr T. Friston K. J. (2020). A bayesian account of generalist and specialist formation under the active inference framework. Front. Artif. Intell. 3:69. 10.3389/frai.2020.00069 33733186 PMC7861269

[B19] Conway J. (2001). Sensory-perceptual episodic memory and its context: autobiographical memory. Philos. Trans. R. S. Lond. B. Biol. Sci. 356, 1375–1384. 10.1098/rstb.2001.0940 11571029 PMC1088521

[B20] Craik K. J. W. (1952). The Nature of Explanation. Cambridge: Cambridge University Press.

[B21] Da Costa L. Parr T. Sajid N. Veselic S. Neacsu V. Friston K. (2020). Active inference on discrete state-spaces: a synthesis. J. Math. Psychol. 99:102447. 10.1016/j.jmp.2020.102447 33343039 PMC7732703

[B23] Da Costa L. Parr T. Sengupta B. Friston K. (2021). Neural dynamics under active inference: plausibility and efficiency of information processing. Entropy (Basel) 23:454. 10.3390/e23040454 33921298 PMC8069154

[B24] Dauwels J. (2007). “On variational message passing on factor graphs,” in 2007 IEEE International Symposium on Information Theory, (Nice: IEEE), 2546–2550.

[B25] David A. S. (1990). Insight and psychosis. Br. J. Psychiatry 156, 798–808. 10.1192/bjp.156.6.798 2207510

[B26] Eichenbaum H. Sauvage M. Fortin N. Komorowski R. Lipton P. (2012). Towards a functional organization of episodic memory in the medial temporal lobe. Neurosci. Biobehav. Rev. 36, 1597–1608. 10.1016/j.neubiorev.2011.07.006 21810443 PMC3227798

[B27] Fiser J. Berkes P. Orbán G. Lengyel M. (2010). Statistically optimal perception and learning: from behavior to neural representations. Trends Cogn. Sci. 14, 119–130. 10.1016/j.tics.2010.01.003 20153683 PMC2939867

[B28] Fleming S. (2021). Know Thyself: How the New Science of Self Awareness Gives Us the Edge. London: John Murray Press.

[B29] Fleming S. M. Dolan R. J. (2012). The neural basis of metacognitive ability. Philos. Trans. R. S. B. Biol. Sci. 367, 1338–1349. 10.1098/rstb.2011.0417 22492751 PMC3318765

[B30] Fleming S. M. Ryu J. Golfinos J. G. Blackmon K. E. (2014). Domain-specific impairment in metacognitive accuracy following anterior prefrontal lesions. Brain 137, 2811–2822. 10.1093/brain/awu221 25100039 PMC4163038

[B31] Fleming S. M. Weil R. S. Nagy Z. Dolan R. J. Rees G. (2010). Relating introspective accuracy to individual differences in brain structure. Science 329, 1541–1543. 10.1126/science.1191883 20847276 PMC3173849

[B32] Foster D. J. (2017). Replay comes of age. Annu. Rev. Neurosci. 40, 581–602. 10.1146/annurev-neuro-072116-031538 28772098

[B33] Foster D. J. Wilson M. A. (2007). Hippocampal theta sequences. Hippocampus 17, 1093–1099. 10.1002/hipo.20345 17663452

[B34] Fotopoulou A. (2012). Illusions and delusions in anosognosia for hemiplegia: from motor predictions to prior beliefs. Brain 135, 1344–1346. 10.1093/brain/aws094 22532091

[B35] Friston K. Buzsaki G. (2016). The functional anatomy of time: what and when in the brain. Trends Cogn. Sci. 20, 500–511. 10.1016/j.tics.2016.05.001 27261057

[B36] Friston K. Kiebel S. (2009). Predictive coding under the free-energy principle. Philos. Trans. R. Soc. Lond. B. Biol. Sci. 364, 1211–1221. 10.1098/rstb.2008.0300 19528002 PMC2666703

[B37] Friston K. Da Costa L. Hafner D. Hesp C. Parr T. (2021). Sophisticated inference. Neural Comput. 33, 713–763. 10.1162/neco_a_01351 33626312

[B38] Friston K. FitzGerald T. Rigoli F. Schwartenbeck P. Pezzulo G. (2017). Active inference: a process theory. Neural Comput. 29, 1–49. 10.1162/NECO_a_00912 27870614

[B39] Friston K. J. Lin M. Frith C. D. Pezzulo G. Hobson J. A. Ondobaka S. (2017a). Active inference, curiosity and insight. Neural Comput. 29, 2633–2683. 10.1162/neco_a_00999 28777724

[B40] Friston K. J. Rosch R. Parr T. Price C. Bowman H. (2017b). Deep temporal models and active inference. Neurosci. Biobehav. Rev. 77, 388–402. 28416414 10.1016/j.neubiorev.2017.04.009 PMC5461873

[B41] Friston K. J. Parr T. Yufik Y. Sajid N. Price C. J. Holmes E. (2020). Generative models, linguistic communication and active inference. Neurosci. Biobehav. Rev. 118, 42–64. 32687883 10.1016/j.neubiorev.2020.07.005 PMC7758713

[B42] Friston K. Rigoli F. Ognibene D. Mathys C. Fitzgerald T. Pezzulo G. (2015). Active inference and epistemic value. Cogn. Neurosci. 6, 187–214. 10.1080/17588928.2015.1020053 25689102

[B43] Friston K. Schwartenbeck P. FitzGerald T. Moutoussis M. Behrens T. Dolan R. J. (2014). The anatomy of choice: dopamine and decision-making. Philos. Trans. R. Soc. Lond. B. Biol. Sci. 369:20130481. 10.1098/rstb.2013.0481 25267823 PMC4186234

[B44] Funahashi S. Bruce C. J. Goldman-Rakic P. S. (1989). Mnemonic coding of visual space in the monkey’s dorsolateral prefrontal cortex. J. Neurophysiol. 61, 331–349. 10.1152/jn.1989.61.2.331 2918358

[B45] Gärdenfors P. (2005). “The detachment of thought,” in The Mind as a Scientific Object: Between Brain and Culture, eds Erneling C. E. Johnson D. M. (New York, NY: Oxford University Press), 323–341.

[B46] Gregory R. L. (1980). Perceptions as hypotheses. Philos. Trans. R. Soc. Lond. B. Biol. Sci. 290, 181–197. 10.1098/rstb.1980.0090 6106237

[B47] Heins R. C. Mirza M. B. Parr T. Friston K. Kagan I. Pooresmaeili A. (2020). Deep active inference and scene construction. Front. Artif. Intell. 3:509354. 10.3389/frai.2020.509354 33733195 PMC7861336

[B48] Helmholtz H. V. (1866). “Concerning the perceptions in general,” in Treatise on Physiological Optics, ed. Southall J. P. C. (New York: Dover), 1–37.

[B49] Hesp C. Smith R. Parr T. Allen M. Friston K. J. Ramstead M. J. D. (2021). Deeply felt affect: the emergence of valence in deep active inference. Neural Comput. 33, 398–446. 10.1162/neco_a_01341 33253028 PMC8594962

[B50] Hohwy J. (2016). The self-evidencing brain. Noûs 50, 259–285. 10.1111/nous.12062

[B51] Hutsler J. J. (2003). The specialized structure of human language cortex: pyramidal cell size asymmetries within auditory and language-associated regions of the temporal lobes. Brain Lang. 86, 226–242. 10.1016/s0093-934x(02)00531-x 12921766

[B52] Jadhav S. P. Kemere C. German P. W. Frank L. M. (2012). Awake hippocampal sharp-wave ripples support spatial memory. Science 336, 1454–1458. 10.1126/science.1217230 22555434 PMC4441285

[B53] Kier E. L. Staib L. H. Davis L. M. Bronen R. A. (2004). MR imaging of the temporal stem: anatomic dissection tractography of the uncinate fasciculus, inferior occipitofrontal fasciculus and meyer’s loop of the optic radiation. Am. J. Neuroradiol. 25, 677–691. 15140705 PMC7974480

[B54] Kilteni K. Andersson B. J. Houborg C. Ehrsson H. H. (2018). Motor imagery involves predicting the sensory consequences of the imagined movement. Nat. Commun. 9:1617. 10.1038/s41467-018-03989-0 29691389 PMC5915435

[B55] Korsakoff S. (1887). Disturbance of psychic function in alcoholic paralysis and its relation to the disturbance of the psychic sphere in multiple neuritis of non-alcoholic origin. Vestnik Psichiatrii 4, 1–102.

[B56] Kounios J. Beeman M. (2014). The cognitive neuroscience of insight. Annu. Rev. Psychol. 65, 71–93. 10.1146/annurev-psych-010213-115154 24405359

[B57] Lipton P. (2017). “Inference to the best explanation,” in A Companion to the Philosophy of Science, ed. Newton-Smith W. H. (Oxford/Malden, MA: Blackwell), 184–193. 10.1002/9781405164481.ch29

[B58] Louie K. Wilson M. A. (2001). Temporally structured replay of awake hippocampal ensemble activity during rapid eye movement sleep. Neuron 29, 145–156. 10.1016/s0896-6273(01)00186-6 11182087

[B59] Mahr J. B. Csibra G. (2018). Why do we remember? The communicative function of episodic memory. Behav. Brain Sci. 41, 1–93. 10.1017/S0140525X17000012 28100294 PMC5404722

[B60] Maisto D. Friston K. Pezzulo G. (2019). Caching mechanisms for habit formation in active inference. Neurocomputing 359, 298–314. 10.1016/j.neucom.2019.05.083 32055104 PMC7001981

[B61] Mirza M. B. Adams R. A. Mathys C. Friston K. J. (2018). Human visual exploration reduces uncertainty about the sensed world. PLoS One 13:e0190429. 10.1371/journal.pone.0190429 29304087 PMC5755757

[B62] Naito A. Kita H. (1994). The cortico-pallidal projection in the rat: an anterograde tracing study with biotinylated dextran amine. Brain Res. 653, 251–257. 10.1016/0006-8993(94)90397-2 7526961

[B63] Nambu A. (2004). A new dynamic model of the cortico-basal ganglia loop. Prog. Brain Res. 143, 461–466. 10.1016/S0079-6123(03)43043-4 14653188

[B64] O’Keefe J. Dostrovsky J. (1971). The hippocampus as a spatial map: preliminary evidence from unit activity in the freely-moving rat. Brain Res. 34, 171–175. 10.1016/0006-8993(71)90358-1 5124915

[B65] Paolucci C. (2021). “Perception as controlled hallucination,” in Cognitive Semiotics: Integrating Signs, Minds, Meaning and Cognition, ed. Paolucci C. (Cham: Springer), 127–157.

[B66] Parr T. Friston K. J. (2017). Working memory, attention and salience in active inference. Sci. Rep. 7:14678. 10.1038/s41598-017-15249-0 29116142 PMC5676961

[B67] Parr T. Limanowski J. Rawji V. Friston K. (2021). The computational neurology of movement under active inference. Brain 144, 1799–1818. 10.1093/brain/awab085 33704439 PMC8320263

[B68] Parr T. Markovic D. Kiebel S. J. Friston K. J. (2019). Neuronal message passing using mean-field, bethe and marginal approximations. Sci. Rep. 9:1889. 10.1038/s41598-018-38246-3 30760782 PMC6374414

[B69] Parr T. Pezzulo G. Friston K. J. (2022). Active Inference: The Free Energy Principle in Mind, Brain and Behavior. Cambridge, MA: The MIT Press.

[B70] Pearl J. (2009). Causal inference in statistics: an overview. Statist. Surv. 3, 96–146. 10.1214/09-SS057

[B71] Pearl J. (2010). An introduction to causal inference. Int. J. Biostat. 6:7. 10.2202/1557-4679.1203 20305706 PMC2836213

[B72] Pezzulo G. Castelfranchi C. (2007). The symbol detachment problem. Cogn. Process. 8, 115–131. 10.1007/s10339-007-0164-0 17406918

[B73] Pezzulo G. Kemere C. van der Meer M. A. A. (2017). Internally generated hippocampal sequences as a vantage point to probe future-oriented cognition. Ann. N. Y. Acad. Sci. 1396, 144–165. 10.1111/nyas.13329 28548460

[B74] Pezzulo G. Rigoli F. Chersi F. (2013). The mixed instrumental controller: using value of information to combine habitual choice and mental simulation. Front. Psychol. 4:92. 10.3389/fpsyg.2013.00092 23459512 PMC3586710

[B75] Pezzulo G. Rigoli F. Friston K. J. (2018). Hierarchical active inference: a theory of motivated control. Trends Cogn. Sci. 22, 294–306. 10.1016/j.tics.2018.01.009 29475638 PMC5870049

[B76] Pezzulo G. Zorzi M. Corbetta M. (2021). The secret life of predictive brains: what’s spontaneous activity for? Trends Cogn. Sci. 25, 730–743. 10.1016/j.tics.2021.05.007 34144895 PMC8363551

[B77] Price C. J. (2000). The anatomy of language: contributions from functional neuroimaging. J. Anat. 197, 335–359. 10.1046/j.1469-7580.2000.19730335.x 11117622 PMC1468137

[B78] Psillos S. (2002). Causation and Explanation. Netherlands: Acumen Publishing.

[B79] Rikhye R. V. Gilra A. Halassa M. M. (2018). Thalamic regulation of switching between cortical representations enables cognitive flexibility. Nat. Neurosci. 21, 1753–1763. 10.1038/s41593-018-0269-z 30455456 PMC7225728

[B80] Sajid N. Ball P. J. Parr T. Friston K. J. (2021). Active inference: demystified and compared. Neural Comput. 33, 674–712. 10.1162/neco_a_01357 33400903

[B81] Shadlen M. N. Newsome W. T. (1996). Motion perception: seeing and deciding. Proc. Natl. Acad. Sci. U S A 93, 628–633. 10.1073/pnas.93.2.628 8570606 PMC40102

[B82] Shea N. Boldt A. Bang D. Yeung N. Heyes C. Frith C. D. (2014). Supra-personal cognitive control and metacognition. Trends Cogn. Sci. 18, 186–193. 10.1016/j.tics.2014.01.006 24582436 PMC3989995

[B83] Shipp S. Adams R. A. Friston K. J. (2013). Reflections on agranular architecture: predictive coding in the motor cortex. Trends Neurosci. 36, 706–716. 10.1016/j.tins.2013.09.004 24157198 PMC3858810

[B84] Smith R. Parr T. Friston K. J. (2019). Simulating emotions: an active inference model of emotional state inference and emotion concept learning. Front. Psychol. 10:2844. 10.3389/fpsyg.2019.02844 31920873 PMC6931387

[B85] Squire L. R. Zola S. M. (1998). Episodic memory, semantic memory and amnesia. Hippocampus 8, 205–211. 10.1002/(SICI)1098-1063(1998)8:3<205::AID-HIPO3>3.0.CO;2-I 9662135

[B86] Stoianov I. Maisto D. Pezzulo G. (2021). The hippocampal formation as a hierarchical generative model supporting generative replay and continual learning. BioRxiv [Preprint]. 10.1101/2020.01.16.908889 35870678

[B87] Tschantz A. Seth A. K. Buckley C. L. (2020). Learning action-oriented models through active inference. PLoS Comput. Biol. 16:e1007805. 10.1371/journal.pcbi.1007805 32324758 PMC7200021

[B88] Turner M. S. Cipolotti L. Yousry T. A. Shallice T. (2008). Confabulation: damage to a specific inferior medial prefrontal system. Cortex 44, 637–648. 10.1016/j.cortex.2007.01.002 18472034

[B89] Ueltzhöffer K. (2018). Deep active inference. Biol. Cybern. 112, 547–573. 10.1007/s00422-018-0785-7 30350226

[B90] Villena-González M. López V. Rodríguez E. (2016). Orienting attention to visual or verbal/auditory imagery differentially impairs the processing of visual stimuli. Neuroimage 132, 71–78. 10.1016/j.neuroimage.2016.02.013 26876471

[B91] Windt J. M. Harkness D. L. Lenggenhager B. (2014). Tickle me, I think I might be dreaming! Sensory attenuation, self-other distinction and predictive processing in lucid dreams. Front. Hum. Neurosci. 8:717. 10.3389/fnhum.2014.00717 25278861 PMC4166313

[B92] Winn J. Bishop C. M. (2005). Variational message passing. J. Mach. Learn. Res. 6, 661–694.

[B93] Yufik Y. M. (2018). “GNOSTRON: a framework for human-like machine understanding,” in 2018 IEEE Symposium Series on Computational Intelligence (SSCI) (Bengaluru, India: IEEE), 136–145.

[B94] Yufik Y. M. Sheridan T. B. (1996). Virtual networks: new framework for operator modeling and interface optimization in complex supervisory Control systems. Ann. Rev. Control 20, 179–195.

